# Demise of the British and Foreign Medical Review

**Published:** 1848-01

**Authors:** 


*Demise of the British and Foreign Medical Review.*—Dr. Forbes has retired from the editorial chair, and the Review formerly conducted by him is to be merged in the Medico-Chirurgical Review, which is henceforth to be entitled *British and Foreign Medico-Chirurgical Review.* It is to be republished by the Woods, of New-York, who have for many years republished the Medico-Chirurgical.

Dr. Forbes, in his valedictory, gives a detailed account of the rise and fail of this enterprise. It would appear that he was led to originate the Review from a painful conviction of the deplorable and degraded condition of medical periodical literature. He speaks of contemporary British Journals (condescending to except from a sweeping expression of contempt a single publication emanating from Dublin) in terms, which display an astonishing degree of egotism and arrogance. It seems that very few articles which appeared in his Review, were from his own pen. Of late, however, he contributed Reviews of homœopathy and hydropathy, which he acknowledges affected injuriously the circulation of the work. As a pecuniary speculation the enterprise was a failure, and as it promised to bring him in debt more and more each year of its continuence, it was prudently abandoned.

We cannot but think that with a little modesty of demeanor Dr. F. would have retired more gracefully. There are some insinuations contained in his valedictory which are unjust in themselves, and certainly are as much in violation of medical ethics as any other species of defamation of the character of his professional brethren. But it is to be recollected that he vacates the editorial chair, not willingly, but with a reluctant concession to the force of circumstances.

